# Association of red blood cell distribution width with hospital admission and in-hospital mortality across all-cause adult emergency department visits

**DOI:** 10.1093/jamiaopen/ooad053

**Published:** 2023-07-13

**Authors:** Woo Suk Hong, Akos Rudas, Elijah J Bell, Jeffrey N Chiang

**Affiliations:** Department of Emergency Medicine, University of California Los Angeles David Geffen School of Medicine, Los Angeles, California, USA; Department of Computational Medicine, University of California Los Angeles David Geffen School of Medicine, Los Angeles, California, USA; Department of Emergency Medicine, University of California Los Angeles David Geffen School of Medicine, Los Angeles, California, USA; Department of Computational Medicine, University of California Los Angeles David Geffen School of Medicine, Los Angeles, California, USA

**Keywords:** red blood cell distribution width, triage, emergency medicine, in-hospital mortality, machine learning, predictive modeling

## Abstract

**Objectives:**

To test the association between the initial red blood cell distribution width (RDW) value in the emergency department (ED) and hospital admission and, among those admitted, in-hospital mortality.

**Materials and Methods:**

We perform a retrospective analysis of 210 930 adult ED visits with complete blood count results from March 2013 to February 2022. Primary outcomes were hospital admission and in-hospital mortality. Variables for each visit included demographics, comorbidities, vital signs, basic metabolic panel, complete blood count, and final diagnosis. The association of each outcome with the initial RDW value was calculated across 3 age groups (<45, 45–65, and >65) as well as across 374 diagnosis categories. Logistic regression (LR) and XGBoost models using all variables excluding final diagnoses were built to test whether RDW was a highly weighted and informative predictor for each outcome. Finally, simplified models using only age, sex, and vital signs were built to test whether RDW had additive predictive value.

**Results:**

Compared to that of discharged visits (mean [SD]: 13.8 [2.03]), RDW was significantly elevated in visits that resulted in admission (15.1 [2.72]) and, among admissions, those resulting in intensive care unit stay (15.3 [2.88]) and/or death (16.8 [3.25]). This relationship held across age groups as well as across various diagnosis categories. An RDW >16 achieved 90% specificity for hospital admission, while an RDW >18.5 achieved 90% specificity for in-hospital mortality. LR achieved a test area under the curve (AUC) of 0.77 (95% confidence interval [CI] 0.77–0.78) for hospital admission and 0.85 (95% CI 0.81–0.88) for in-hospital mortality, while XGBoost achieved a test AUC of 0.90 (95% CI 0.89–0.90) for hospital admission and 0.96 (95% CI 0.94–0.97) for in-hospital mortality. RDW had high scaled weights and information gain for both outcomes and had additive value in simplified models predicting hospital admission.

**Discussion:**

Elevated RDW, previously associated with mortality in myocardial infarction, pulmonary embolism, heart failure, sepsis, and COVID-19, is associated with hospital admission and in-hospital mortality across all-cause adult ED visits. Used alone, elevated RDW may be a specific, but not sensitive, test for both outcomes, with multivariate LR and XGBoost models showing significantly improved test characteristics.

**Conclusions:**

RDW, a component of the complete blood count panel routinely ordered as the initial workup for the undifferentiated patient, may be a generalizable biomarker for acuity in the ED.

## INTRODUCTION 

Recent studies have identified red blood cell distribution width (RDW) as a candidate for a generalizable biomarker for acuity in the emergency department (ED). As a standard component of the complete blood count panel, RDW is defined as the standard deviation (SD) of the red blood cell size over its mean and measures the relative variation of red blood cell size, also called anisocytosis. Formerly limited to studies of cardiovascular disease,^1–13^ elevated RDW has now been associated with adverse outcomes in various disease states, including sepsis,^14–17^ pneumonia,^18^ endocarditis,^19^ chronic obstructive pulmonary disease,^20^ pulmonary embolism,^21^ pancreatitis,^22^ end-stage renal disease,^23^^,^^24^ and, most recently, COVID-19.^25–28^ Elevated RDW has been shown to be a predictor of mortality in the intensive care unit (ICU),^29^^,^^30^ across all hospitalized patients,^31^ and even in the general population,^32^^,^^33^ and outperformed scoring systems such as APACHE and SOFA.^15^

Machine learning models built on large datasets from the electronic health record have also identified RDW as an important predictor of patient outcomes. RDW had high information gain in a random forest model predicting in-hospital mortality in patients with sepsis,^34^ as well as in XGBoost models predicting hospital admission from the ED,^35^ and was a highly weighted predictor in a multivariate logistic regression (LR) model predicting 6-month mortality in older adults presenting to the ED.^36^ These findings have been reproduced in the Medical Information Mart for Intensive Care (MIMIC) IV dataset, with RDW being a predictor of 30-day mortality in older adults with sepsis and 28-day mortality in patients who have combined heart failure and hypertension.^37^^,^^38^

Mechanisms for RDW elevation in disease states have been proposed but not validated.^39^ It has been suggested that anisocytosis reflects impairment in red blood cell production or clearance, secondary to diminished titers and sensitivity to erythropoietin.^40^^,^^41^ RDW has been shown to be elevated in the setting of oxidative stress and to correlate with markers of acute inflammation, such as C-reactive protein and erythrocyte sedimentation rate, as well as with age and telomere length.^42–45^ While elevated RDW may primarily be a marker of underlying risk factors, anisocytosis may also have a direct pathogenic role in cardiovascular disease by impairing capillary blood flow.^46^

Few studies have tested the prognostic value of RDW in the ED. Prior studies have limited their focus on specific disease states, such as acute coronary syndrome,^47^^,^^48^ deep vein thrombosis,^49^ and sepsis,^50^ using small sample sizes ranging from hundreds to thousands of patients. Given the association between RDW and mortality across various disease states, we hypothesized that RDW may be a generalizable biomarker for acuity across all-cause adult ED visits.

## OBJECTIVES

In a large retrospective study of 210 930 all-cause adult ED visits in an academic hospital system, we test the association between the initial RDW value in the ED and hospital admission and, in those admitted to the hospital, in-hospital mortality, as well as test the importance of RDW in machine learning models predicting each outcome.

## MATERIALS AND METHODS

### Patients and study design

The study followed the TRIPOD reporting guideline.^51^ The study used de-identified electronic health records available as preprocessed structured data from the authors’ institution. Personal identifying information had been stripped from the data and the events date-shifted in order to preserve patient privacy. The study did not fall under human subjects research per the IRB at the authors’ institution and further review was deferred.

The dataset included all adult ED visits from March 2013 to February 2022 at a tertiary hospital system comprised of 2 academic centers. Multiple visits per patient were allowed in the dataset. The represented EDs include a level 1 trauma center with an annual census of ∼40 000 patients and a community hospital-based department with an annual census of ∼30 000 patients. Both EDs are located within the same city and are part of a single hospital system utilizing the Epic electronic health record (Verona, WI, USA).

Inclusion criteria were patient age ≥18 and availability of complete blood count laboratory result. Visits with disposition not determined by the medical provider (eg, eloped, left without being seen, left against medical advice) were excluded from the study. Visits resulting in transfers to other hospitals were excluded from the study given the inability to track mortality outcomes.

Primary outcomes were hospital admission and, in those admitted to the hospital, in-hospital mortality. Out-of-hospital mortality, including unexpected deaths after discharge and deaths of those discharged to hospice care, were not tracked. Admissions were further specified into admissions to the floor versus those to the ICU. Variables for each visit included age, sex, body mass index, comorbidities, triage vital signs, basic metabolic panel, complete blood count, and final diagnosis.

More specifically, vital signs included systolic and diastolic blood pressures, heart rate, respiratory rate, oxygen saturation, and temperature. Laboratory results included a complete blood count panel comprised of hemoglobin, RDW, mean corpuscular volume, mean corpuscular hemoglobin, mean corpuscular hemoglobin concentration, white blood cell count, platelet count, and a basic metabolic panel comprised of sodium, potassium, chloride, bicarbonate, blood urea nitrogen, creatinine, and glucose. The initial value was used for vital signs and laboratory results with multiple instances during a single visit, as this was the value least affected by interventions such as transfusion and would yield predictive information at the earliest time. Values above or below a detectable level were assigned the value of the threshold.

Comorbidities listed on the electronic health record were transposed to 27 categories using the Centers for Medicare & Medicaid Services Chronic Conditions Algorithm, an International Classification of Disease (ICD)-based algorithm used by federal agencies for high-level categorization of Medicare beneficiaries.^52^ Final diagnoses listed during the current encounter, including those added by inpatient teams throughout hospitalization, were transposed to ∼500 clinical categories using the Clinical Classifications Software Refined (CCSR) for ICD-10-CM Diagnoses from the Agency for Healthcare Research and Quality.^53^

### Statistical analysis

The mean RDW was calculated by age and by total number of comorbidities. Risk ratios were calculated for each outcome for an elevated RDW level, defined in the authors’ healthcare system as >15.5. The Wilcoxon signed-rank test was used to compare the mean RDW between ED discharges and hospital admissions and, in those admitted to the hospital, between floor and ICU admissions, as well as between survivors and non-survivors.

Given potential confounding from known association between RDW and age, the mean RDW across outcomes was compared within 3 age groups, binned to approximately equal number of samples: <45, 45–65, and >65.

Similarly, subgroup analysis comparing the mean RDW across outcomes was done for each CCSR final diagnosis category containing more than a hundred samples, and the results visualized for the 10 diagnoses with the highest mean RDW, the 10 most frequent diagnoses, and 5 preselected diagnoses of interest: myocardial infarction, pulmonary embolism, COVID-19, heart failure, and sepsis. Visits with diagnoses spanning multiple categories were included in every listed category. Bonferroni method was used to correct for multiple comparisons.

LR and XGBoost models predicting hospital admission were built on all available visits, while those predicting in-hospital mortality were built only on visits that resulted in hospital admission. Final diagnoses were excluded from model training as they were entered throughout a patient’s hospital stay and not available at the time of ED evaluation. All other variables were included. For LR, all categorical variables were converted to numeric variables using one-hot encoding, scaled to the interval between 0 and 1, and missing values imputed using the median. No scaling or imputation was performed for XGBoost, since it learns a default direction for each split in the case that the variable needed for the split is missing.

For both datasets, samples were randomly split into a training set of 80%, a validation set of 10%, and a holdout test set of 10%. Given the lack of hyperparameters, LR models were trained on all samples excluding the test set. For XGBoost, hyperparameters were optimized on the training set by maximizing the area under the curve (AUC) of the validation set via 5-fold cross-validation. The optimized set of hyperparameters was then used to train the XGBoost model on all samples excluding the test set. The final AUC of each model was calculated on the holdout test set with 95% confidence intervals (CIs) constructed using DeLong’s method. Each variable’s scaled weight was extracted from the LR models and ranked by absolute value, while its information gain, a metric that quantifies the improvement in accuracy of a tree-based algorithm from a split based on a given variable, was averaged from a hundred iterations of XGBoost.

Finally, simplified models predicting each outcome using only age, sex, and vital signs were built to test whether RDW had any additive value on the AUC. The model fitting protocol is provided in the Supplementary Methods. All processing and analysis were done in R.

## RESULTS

### Characteristics of visits

Of the 540 889 adult ED visits in the study period, 23 998 visits (4.4%) were excluded due to a disposition not determined by the medical provider (eg, eloped, left without being seen, left against medical advice) and 13 059 visits (2.4%) excluded due to transfers to other hospitals. Of the remaining 503 832 visits, 210 930 (42%) had a complete blood count result and were included in analysis. The cohort was comprised of 116 736 unique patients, with a median and mean visit frequency of 1 and 1.8. The mean (SD) age of all visits was 55 (21) years. In total, 94 347 visits (45%) resulted in hospital admission, divided into 74 826 (35%) floor admissions and 19 521 (9.3%) ICU admissions. A total of 3159 visits (1.5% of all visits and 3.3% of hospital admissions) resulted in in-hospital mortality. A summary of visit characteristics by outcome is shown in Table 1.

**Table 1.** Visit characteristics by outcome, mean (SD)

Characteristics	All visits (*n* = 210 930)	Discharged (*n* = 116 583)	Admitted (*n* = 94 347)	In-hospital death (*n* = 3159)
Demographics				
Age, years	55.5 (20.7)	50.5 (20.3)	61.6 (19.6)	69.4 (16.9)
Sex (% male)	48	51	45	55
Body mass index	26.2 (6.00)	26.4 (5.94)	25.9 (6.07)	25.6 (6.24)
Total number of comorbidities	3.04 (4.61)	2.4 (4.0)	3.9 (5.1)	3.8 (5.3)
Triage vital signs				
Systolic blood pressure, mm Hg	125 (19.3)	127 (18.8)	124 (19.9)	95.1 (34.8)
Diastolic blood pressure, mm Hg	73.2 (12.8)	75.1 (12.2)	71.0 (13.2)	54.1 (20.8)
Heart rate	77.1 (15.7)	76.7 (13.3)	77.6 (18.2)	42.1 (48.8)
Respiratory rate	18.4 (4.33)	17.9 (3.57)	19.0 (5.05)	21.2 (7.44)
Oxygen saturation, %	97.3 (3.51)	97.8 (2.52)	96.7 (4.35)	93.8 (9.25)
Temperature, F	98.0 (1.38)	98.1 (1.44)	97.9 (1.29)	97.1 (4.44)
Complete blood count panel				
White blood cell count, 10^3^/μL	9.18 (7.89)	8.35 (3.83)	10.2 (10.9)	13.9 (19.7)
Hemoglobin, g/dL	12.5 (2.34)	13.0 (2.01)	11.9 (2.56)	10.9 (2.70)
Platelet count, 10^3^/μL	243 (99.3)	245 (83.2)	239 (116)	214 (134)
Red blood cell distribution width, %	14.3 (2.45)	13.8 (2.03)	15.1 (2.72)	16.8 (3.25)
Mean corpuscular volume, fL	89.9 (7.07)	89.3 (6.42)	90.6 (7.73)	92.9 (9.06)
Mean corpuscular hemoglobin, pg	29.6 (2.78)	29.6 (2.59)	29.6 (3.00)	29.6 (3.21)
Mean corpuscular hemoglobin concentration, g/dL	32.9 (1.54)	33.1 (1.41)	32.6 (1.66)	31.9 (1.92)
Basic metabolic panel				
Sodium, mmol/L	139 (4.40)	139 (3.36)	138 (5.24)	137 (7.31)
Potassium, mmol/L	4.09 (0.786)	4.02 (0.521)	4.19 (1.02)	4.48 (2.09)
Chloride, mmol/L	102 (5.07)	103 (3.97)	101 (5.99)	99.8 (7.75)
Bicarbonate, mmol/L	23.6 (3.70)	23.8 (3.11)	23.4 (4.29)	21.8 (6.32)
Blood urea nitrogen, mg/dL	20.0 (15.6)	16.1 (10.0)	24.1 (19.0)	37.1 (26.6)
Creatinine, mg/dL	1.25 (1.65)	1.02 (1.25)	1.52 (2.01)	1.95 (1.99)
Glucose, mg/dL	131 (68.4)	121 (56.8)	137 (74.4)	160 (101)

### Association between RDW and outcomes

**Figure 1.**
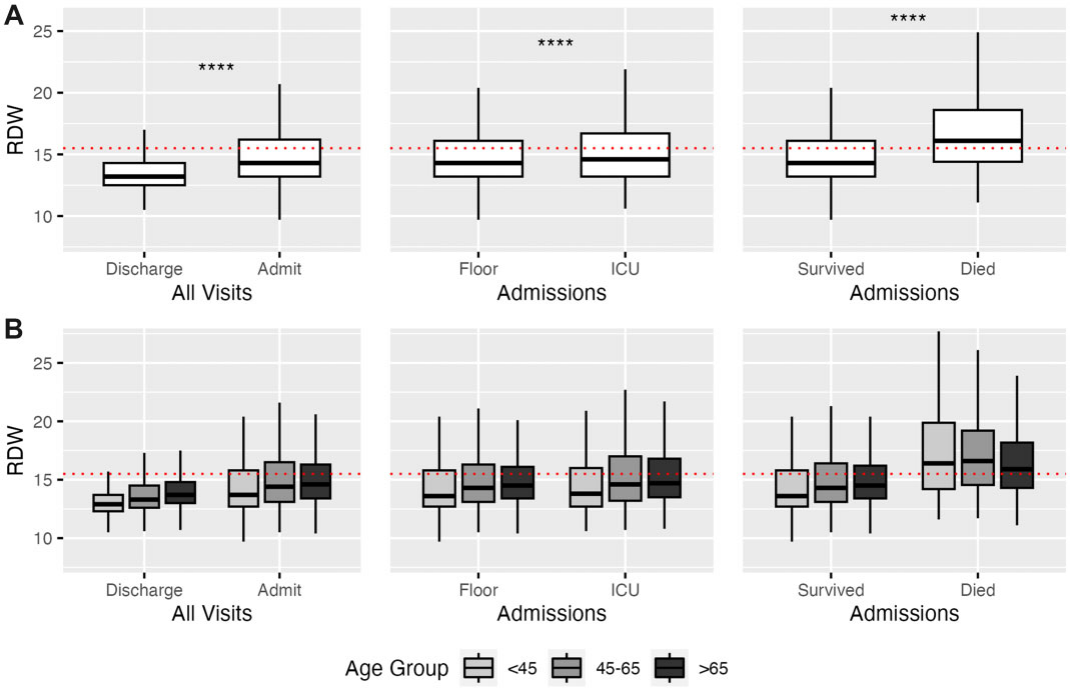
(A) Red blood cell distribution width (RDW) by outcome. All comparisons between means of each binary outcome were significant by Wilcoxon signed-rank test (*P* < 2e−16, denoted by ****). (B) RDW by outcomes by age group. All comparisons between means within each of the 3 age groups were significant by Wilcoxon signed-rank test, with all but Floor versus ICU for age <45 (*P* = 3e−4) and Floor versus ICU for age 45–65 (*P* = 1.3e−13) achieving *P* < 2e−16. Elevated RDW, as defined by the healthcare system (>15.5), is shown by the red dotted line. Thick line represents the median; box represents the interquartile range (IQR); and whiskers represent the 1.5 × IQR from the first and third quartile.

The mean (SD) RDW across all visits was 14.3 (2.45) and increased with age and the cumulative number of comorbidities (Supplementary Figure S1). For all-comers, an elevated RDW level >15.5 had risk ratios of 1.72 (95% CI 1.71–1.74) for hospital admission, 2.06 (95% CI 2.01–2.12) for ICU admission, and 4.87 (95% CI 4.54–5.22) for in-hospital mortality. Compared to that of discharged visits (mean [SD]: 13.8 [2.03]), RDW was significantly elevated in visits that resulted in hospital admission (15.1 [2.72]) and, among admissions, those resulting in ICU stay (15.3 [2.88]) and/or death (16.8 [3.25]). The mean (SD) RDW for the 3 predefined age groups (<45, 45–65, and >65) were 13.8 (2.36), 14.5 (2.50), and 14.8 (2.37), respectively. For all age groups, RDW was significantly elevated in visits that resulted in hospital admission and, among admissions, those resulting in ICU stay and/or death (Figure 1, Table 2).

**Table 2.** Red blood cell distribution width, mean (SD), by outcome by age group

Age	Discharged	Admitted				
<45 *n* = 69 869	13.4 (1.95) *n* = 49 812	14.7 (2.96) *n* = 20 057	Floor	14.7 (2.94) *n* = 16 333	Survived	14.6 (2.93) *n* = 19 763
			ICU	14.8 (3.06) *n* = 3724	Died	17.3 (3.93) *n* = 294
45–65 *n* = 66 034	13.9 (2.04) *n* = 35 921	15.2 (2.80) *n* = 30 113	Floor	15.1 (2.74) *n* = 23 557	Survived	15.1 (2.76) *n* = 29 250
			ICU	15.4 (3.01) *n* = 6556	Died	17.2 (3.44) *n* = 863
>65 *n* = 75 027	14.2 (2.01) *n* = 30 850	15.2 (2.52) *n* = 44 177	Floor	15.1 (2.47) *n* = 34 936	Survived	15.1 (2.47) *n* = 42 175
			ICU	15.4 (2.67) *n* = 9241	Died	16.5 (3.03) *n* = 2002
All *n* = 210 930	13.8 (2.03) *n* = 116 583	15.1 (2.72) *n* = 94 347	Floor	15.0 (2.67) *n* = 74 826	Survived	15.0 (2.68) *n* = 91 188
			ICU	15.3 (2.88) *n* = 19 521	Died	16.8 (3.25) *n* = 3159

ICU: intensive care unit.

### RDW by final diagnosis

**Figure 2.**
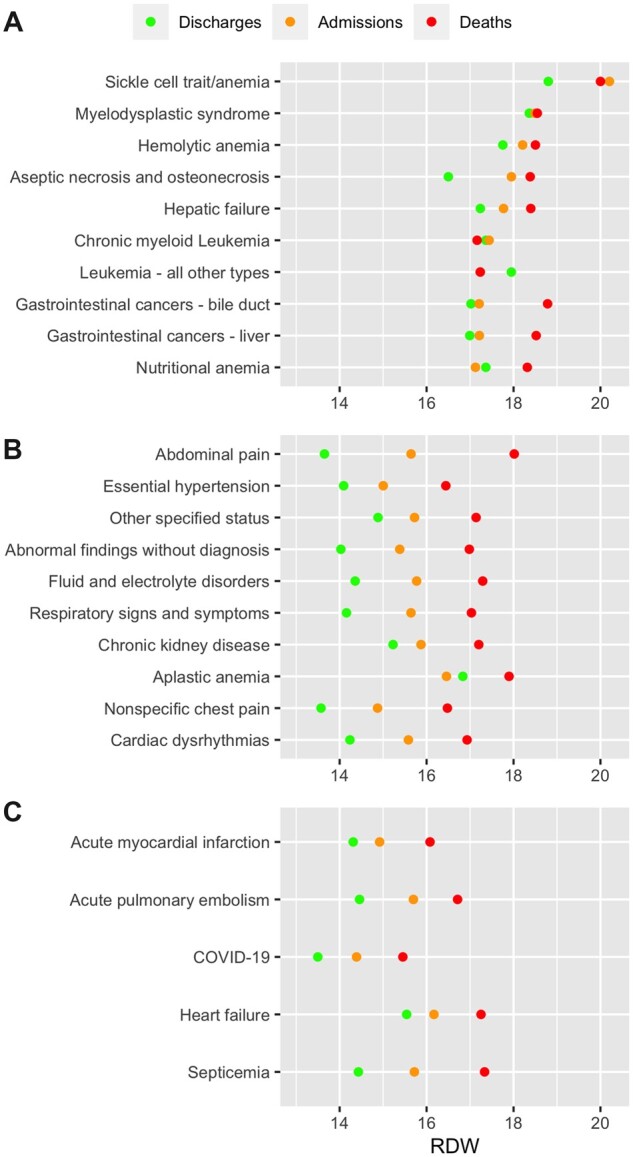
Red blood cell distribution width (RDW) by final diagnosis. (A) Ten diagnoses with the highest mean RDW. (B) Ten most frequent diagnoses. (C) Preselected diagnoses of interest. The mean RDW by outcome for every diagnosis category, as well as its standard deviation and *P*-value for difference across outcomes, is available in Supplementary Table S1.

Of the 542 CCSR diagnosis categories, 509 were included the dataset, and 374 categories retained after excluding those with fewer than a hundred samples. Sickle cell anemia had the highest mean RDW, followed by myelodysplastic syndrome and hemolytic anemia, while abdominal pain was the most frequent diagnosis. Other than in sickle cell anemia for hospital admission and in liver cancer and nutritional anemia for in-hospital mortality, elevated RDW was not associated with either hospital admission or in-hospital mortality in the top 10 diagnoses with the highest mean RDW, while elevated RDW was associated with both hospital admission and in-hospital mortality in the top 10 most frequent diagnoses and the 5 pre-selected diagnoses of interest, namely, myocardial infarction, pulmonary embolism, COVID-19, heart failure, and sepsis (Bonferroni *P* < 1.3e−4) (Figure 2). The mean RDW by outcome for every diagnosis category, as well as its SD and *P*-value for difference across outcomes, is available in Supplementary Table S1.

### Model performance, scaled weight, and information gain

**Figure 3.**
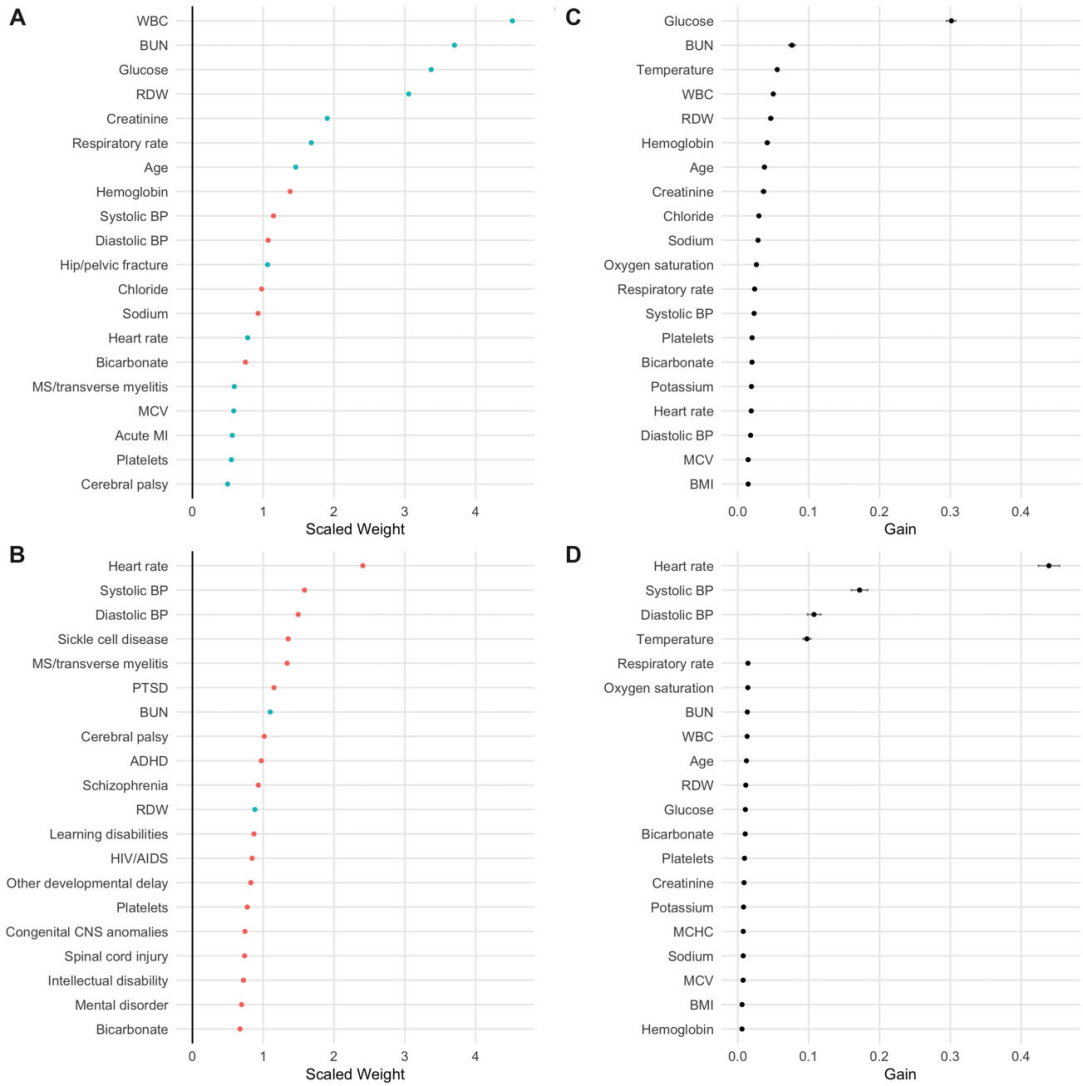
(A) Variables with highest scaled weights by absolute value in logistic regression (LR) model predicting admission. Positive weights are shown in blue, negative in red. Medical conditions represent comorbidities, not final diagnoses. (B) Variables with highest scaled weights by absolute value in LR model predicting in-hospital mortality. (C) Variables with highest information gain in XGBoost model predicting admission. (D) Variables with highest information gain in XGBoost model predicting in-hospital mortality. 95% confidence intervals are shown by gray bars. ADHD: attention-deficit/hyperactivity disorder; BMI: body mass index; BP: blood pressure; BUN: blood urea nitrogen; MCHC: mean corpuscular hemoglobin concentration; MCV: mean corpuscular volume; MI: myocardial infarction; PTSD: post-traumatic stress disorder; RDW: red blood cell distribution width; WBC: white blood cell count.

LR models had a test AUC of 0.77 (95% CI 0.77–0.78) for hospital admission and 0.85 (95% CI 0.81–0.88) for in-hospital mortality. RDW had high scaled weights for both outcomes, while blood urea nitrogen and heart rate had the highest absolute scaled weights, respectively, for predicting admission and in-hospital mortality (Figure 3A and B). XGBoost models had a test AUC value of 0.90 (95% CI 0.89–0.90) for hospital admission and 0.96 (95% CI 0.94–0.97) for in-hospital mortality. RDW had high information gain for both outcomes, while blood glucose level and heart rate had the highest information gain, respectively, for predicting admission and in-hospital mortality (Figure 3C and D). The sensitivity, specificity, positive predictive value, and negative predictive value of each model, compared to univariate models using RDW alone, are shown in Table 3.

**Table 3.** Test characteristics of models

Outcome	Model	Sensitivity	Specificity	PPV	NPV
Admission	RDW > 15.5	0.32	0.87	0.67	0.61
	RDW > 16	0.27	0.90	0.68	0.60
	LR	0.41	0.90	0.76	0.66
	XGBoost	0.65	0.90	0.83	0.76
In-hospital mortality	RDW > 15.5	0.57	0.69	0.06	0.98
	RDW > 18.5	0.25	0.90	0.08	0.97
	LR	0.67	0.90	0.19	0.99
	XGBoost	0.89	0.90	0.23	0.99

Apart from the univariate model RDW >15.5, the cutoff threshold for every model was set to achieve a specificity of 90%. 95% confidence intervals, calculated using the Clopper Pearson method, were <±0.01.

AUC: area under the curve; LR: logistic regression; NPV: negative predictive value; PPV: positive predictive value; RDW: red blood cell distribution width.

Simplified models predicting hospital admission using only age, sex, and vital signs showed increases in AUC from 0.68 (95% CI 0.67–0.69) to 0.71 (95% CI 0.70–0.72) for LR and 0.75 (95% CI 0.75–0.76) to 0.77 (95% CI 0.76–0.78) for XGBoost with the addition of RDW, while RDW had no significant additive value in simplified models predicting in-hospital mortality (Supplementary Figure S2). Scaled weights and information gain for all variables, as well as their bootstrapped CIs, are available in Supplementary Tables S2–S5.

## DISCUSSION

In a large study of 210 930 adult ED visits, we show that an elevated RDW is significantly associated with hospital admission and in-hospital mortality across all across all age groups. RDW had high scaled weights and information gain for both outcomes in LR and XGBoost models that used demographics, comorbidities, vital signs, complete blood count, and basic metabolic panel, and showed additive value in simplified models predicting hospital admission.

While most previous studies have limited their focus on specific diseases, this study confirms that elevated RDW is a marker of risk for hospitalization and death across a general adult ED population, as well as reproducing findings from previous studies documenting association between elevated RDW and mortality in myocardial infarction,^1^^,^^3^^,^^5^^,^^7^^,^^47^^,^^48^ pulmonary embolism,^20^^,^^21^ COVID-19,^25–28^ heart failure,^8–13^ and sepsis.^14^^,^^16^^,^^17^ RDW showed high discriminatory value in risk-stratifying common diagnoses such as abdominal pain and hypertension, as well as vague, nonspecific categories such as “Other specified status” and “Abnormal findings without diagnosis,” highlighting its relevance in various disease states beyond cardiovascular disease.

While RDW was the highest in sickle cell disease, hemolytic anemia, and hematologic malignancies, it was in these disease states where RDW was least associated with in-hospital mortality. High baseline RDW due to pathologic erythropoiesis and frequent transfusions causing iatrogenic elevations may obscure the association in hematologic diseases^54–56^ compared to other settings where RDW elevation may reflect an aggregated comorbidity burden, though others have found elevated RDW to be associated with poor outcomes even in hematologic malignancies.^57^ RDW was also markedly elevated in hepatic failure and hepatobiliary malignancies, highlighting the role of the liver in potential mechanisms for RDW elevation, including impaired red blood cell clearance, diminished erythropoiesis, and nutritional deficiencies from underlying alcoholism.^58–62^

We identify several other predictors for hospital admission and in-hospital mortality. In concordance with prior literature, vital signs were highly predictive of both outcomes, with the heart rate and blood pressure having the highest absolute scaled weights and information gain for in-hospital mortality.^63–65^ Laboratory tests with higher absolute scaled weight and information gain than RDW included white blood cell count and blood urea nitrogen, both of which have been extensively shown to be associated with poor outcomes in various diseases.^66–71^

Despite the mounting evidence on the association between RDW and poor outcomes, the role of RDW in clinical decision-making remains ambiguous. Used alone, elevated RDW may be a specific, but not sensitive, test for acuity. In this study, RDW >16 achieved 90% specificity for hospital admission and RDW >18.5 achieved 90% specificity for in-hospital mortality. Although multivariate LR and XGBoost models showed significantly improved test characteristics as expected for machine learning models leveraging large datasets,^72^ such models often do not aid the provider’s clinical decision-making unless integrated automatically into the electronic health record, and even when integrated as clinical decision support, have not been shown to result in higher quality care.^73–75^ Simplified models using only age, sex, and vital signs showed a modest, but significant, increase in AUC in predicting admission when RDW was added, while RDW had no additive value in predicting in-hospital mortality, suggesting that vital signs, which comprised the top 5 variables with the highest information gain for in-hospital mortality, may contain enough information as to render additional variables negligible. Further studies are necessary to establish the clinical utility of RDW, which may be limited to specific chief complaints or age groups.

This study has several limitations. While the average number of visits per patient was low, this study did not control for potential confounding from high utilizers. Using hospital admission as an assay for patient acuity, this study did not take account of patients who were discharged but should have been admitted by objective criteria. Similarly, patients who did not get a complete blood count panel, or who eloped during their medical evaluation, left against medical advice, or left without being seen by a provider were excluded from the study. Additionally, visits that resulted in transfers to other hospitals were also excluded. The study also excluded out-of-hospital mortality or deaths that occurred shortly after hospital discharge, such as those of patients discharged to hospice care. The study only used the initial RDW value recorded during the visit: changes in value across time and in response to blood product transfusions or other treatments, whether in the ED or in the inpatient setting, and their potential effects on outcomes, were not explored. Finally, the study was conducted in a hospital system located in the same geographic region and may not be generalizable to a larger population.

## CONCLUSION

Noninvasive and easily measured, RDW is part of the complete blood count panel that is routinely ordered as the initial workup for the undifferentiated patient. RDW is associated with hospital admission and in-hospital mortality across a general adult ED population, making it a potential candidate as a generalizable biomarker for acuity.

## Supplementary Material

ooad053_Supplementary_Data

## Data Availability

The data underlying this article cannot be shared publicly due to institutional policy.
