# Supplementary material for: Association of red blood cell distribution width with hospital admission and in-hospital mortality across all-cause adult emergency department visits
Source: JAMIA Open. 2023 Jul 13;6(3):ooad053. doi: 10.1093/jamiaopen/ooad053 (PMC10368803; doi:10.1093/jamiaopen/ooad053)
Supplement: ooad053_Supplementary_Data [file ooad053_supplementary_data.zip › rdw_supplement_JAMIA_v2.docx]

**SUPPLEMENT**

Association of Red Blood Cell Distribution Width With Hospital Admission and In-Hospital Mortality Across All-Cause Adult Emergency Department Visits

**Table of Contents**

**Methods Supplement.** Model fitting protocol

**Figure S1.** Mean RDW by A) Age and B) Total number of comorbidities.

**Figure S2.** Additive value of RDW in simplified models using age, sex, and vital signs.

**Table S1.** Mean RDW by outcome by final diagnosis category.

**Table S2.** Scaled weights for LR model predicting hospital admission

**Table S3.** Scaled weights for LR model predicting in-hospital mortality

**Table S4.** Information gain for XGBoost model predicting hospital admission

**Table S5.** Information gain for XGBoost model predicting in-hospital mortality

**Methods Supplement.** Model fitting protocol

Logistic regression can be represented as a neural network with no hidden layer and was implemented accordingly in *keras.* A fixed optimizer setting incorporating regularization (optimizer = rmsprop, learning rate = 0.001) was used to train the models. Note that *keras* optimizes accuracy rather than the area-under-the-curve (AUC). The hyperparameters for the *keras* package (version 2.0.8) can be found in its online documentation ([https://keras.rstudio.com](https://keras.rstudio.com/)).[1] The AUC for each LR model is provided below:

| **Data** | **Outcome** | **Architecture** | **Train Acc** | **Test AUC** | **95% CI Lower** | **95% CI Upper** |
| --- | --- | --- | --- | --- | --- | --- |
| All visits  (n = 210,930),  All variables | Admission  (m = 94,347, 45%) | 98 ⇒ 1  optimizer_rmsprop(lr = 0.001), 5 epochs | 0.71 | 0.77 | 0.77 | 0.78 |
| All visits  (n = 210,930),  Age + Sex + Vitals | Admission  (m = 94,347, 45%) | 8 ⇒ 1  optimizer_rmsprop(lr = 0.001), 5 epochs | 0.64 | 0.68 | 0.67 | 0.69 |
| All visits  (n = 210,930),  Age + Sex + Vitals + RDW | Admission  (m = 94,347, 45%) | 9 ⇒ 1  optimizer_rmsprop(lr = 0.001), 5 epochs | 0.66 | 0.71 | 0.70 | 0.72 |
| Admitted  (n = 94,347),  All variables | In-hospital mortality  (m = 3,159, 3.3%) | 98 ⇒ 1  optimizer_rmsprop(lr = 0.001), 5 epochs | 0.97 | 0.85 | 0.81 | 0.88 |
| Admitted  (n = 94,347),  Age + Sex + Vitals | In-hospital mortality  (m = 3,159, 3.3%) | 8 ⇒ 1  optimizer_rmsprop(lr = 0.001), 5 epochs | 0.96 | 0.83 | 0.79 | 0.86 |
| Admitted  (n = 94,347),  Age + Sex + Vitals + RDW | In-hospital mortality  (m = 3,159, 3.3%) | 9 ⇒ 1  optimizer_rmsprop(lr = 0.001), 5 epochs | 0.96 | 0.85 | 0.81 | 0.88 |

XGBoost is a scalable implementation of gradient boosting, an algorithm that minimizes loss by combining a series of simple linear or tree-based learners.[2,3] XGBoost has a natural way of handling missing values by learning a default direction in the case that the feature needed for the split is missing, and thus is particularly suited for EHR data.

The hyperparameters for the *xgboost* package (version 0.6-4) and their default values are listed in its online documentation (<http://xgboost.readthedocs.io/en/latest/parameter.html>). The following hyperparameters were tuned to maximize the average performance on the validation sets after five-fold cross-validation:

- max_depth (10, 15, 20, 25)
- colsample_by_level (0.1, 0.3)
- nrounds (10, 20)

The following hyperparameters were fixed:

- eta = 0.3
- nthread = 5

All other hyperparameters were left to their default values. In case of a tie up to the 3rd decimal place, preference was given to the lower-complexity model. The optimized set of hyperparameters for each XGBoost model and its AUC is provided below:

| **Data** | **Outcome** | **Hyperparameters** | **Train AUC** | **Val AUC** | **Test AUC** | **95% CI Lower** | **95% CI Upper** |
| --- | --- | --- | --- | --- | --- | --- | --- |
| All visits  (n = 210,930),  All variables | Admission  (m = 94,347, 45%) | max_depth = 15, nrounds = 20, colsample_by_level = 0.1 | 0.95 | 0.90 | 0.90 | 0.89 | 0.90 |
| All visits  (n = 210,930),  Age + Sex + Vitals | Admission  (m = 94,347, 45%) | max_depth = 10, nrounds = 10, colsample_by_level = 0.1 | 0.77 | 0.75 | 0.75 | 0.75 | 0.76 |
| All visits  (n = 210,930),  Age + Sex + Vitals + RDW | Admission  (m = 94,347, 45%) | max_depth = 10, nrounds = 10, colsample_by_level = 0.1 | 0.79 | 0.78 | 0.77 | 0.76 | 0.78 |
| Admitted  (n = 94,347),  All variables | In-hospital mortality  (m = 3,159, 3.3%) | max_depth = 15, nrounds = 10, colsample_by_level = 0.1 | 0.99 | 0.96 | 0.96 | 0.94 | 0.97 |
| Admitted  (n = 94,347),  Age + Sex + Vitals | In-hospital mortality  (m = 3,159, 3.3%) | max_depth = 10, nrounds = 10, colsample_by_level = 0.1 | 0.96 | 0.95 | 0.95 | 0.93 | 0.96 |
| Admitted  (n = 94,347),  Age + Sex + Vitals + RDW | In-hospital mortality  (m = 3,159, 3.3%) | max_depth = 10, nrounds = 10, colsample_by_level = 0.1 | 0.97 | 0.96 | 0.95 | 0.94 | 0.97 |

**REFERENCES**

1 Arnold TB. kerasR: R Interface to the Keras Deep Learning Library. J. Open Source Softw. 2017. doi:10.21105/joss.00296

2 Friedman J. Greedy Function Approximation: A Gradient Boosting Machine. In: *Annals of Statistics*. 2000. 1189–232.http://citeseerx.ist.psu.edu/viewdoc/summary?doi=10.1.1.29.9093 (accessed 9 Feb 2018).

3 Chen T, Guestrin C. XGBoost: A Scalable Tree Boosting System. *ArXiv160302754 Cs* 2016;:785–94. doi:10.1145/2939672.2939785
